# Improved Performance of Composite Bipolar Plates for PEMFC Modified by Homogeneously Dispersed Multi-Walled Carbon Nanotube Networks Prepared by In Situ Chemical Deposition

**DOI:** 10.3390/nano13020365

**Published:** 2023-01-16

**Authors:** Wenkai Li, Zhiyong Xie, Shi Qiu, Haodong Zeng, Minqi Liu, Gangsheng Wu

**Affiliations:** 1 Carbon-Carbon Composite Materials Research Institute of Powder Metallurgy Research Institute, Central South University, Changsha 410017, China; 2 Guangdong Hydrogen Development New Material Technology Co., Ltd., A1 (Block 2), No. 28, Xingsheng East Road, Hecheng Street, Gaoming District, Foshan 528500, China

**Keywords:** multi-walled carbon nanotubes, composite bipolar plates, PEMFC, MWCNTs networks, chemical vapor deposit, conductivity

## Abstract

Composite bipolar plates with excellent performance play a crucial role in improving the overall performance of proton-exchange-membrane fuel cells. However, for graphite/resin composite bipolar plates, their electrical conductivity and mechanical properties are often too complex to meet the needs of users at the same time. Although nanoconductive fillers can alleviate this problem, the performance improvement for composite bipolar plates is often limited due to problems such as agglomeration. In this study, a uniformly dispersed multi-walled carbon nanotube network was prepared by in situ vapor deposition on the surface and pores of expanded graphite, which effectively avoided the problem of agglomeration and effectively improved the various properties of the composite BPs through the synergistic effect with graphite. With the addition of 2% in situ deposited carbon nanotubes, the modified composite bipolar plate has the best conductivity (334.53 S/cm) and flexural strength (50.24 MPa), and all the properties can meet the DOE requirements in 2025. Using the in situ deposition of carbon nanotubes to modify composite bipolar plates is a feasible route because it can result in multi-walled carbon nanotubes in large quantities and avoid the agglomeration phenomenon caused by adding nanofillers. It can also significantly improve the performance of composite bipolar plates, achieving the high performance of composite bipolar plates at a lower cost.

## 1. Introduction

Proton-exchange-membrane fuel cells (PEMFCs) have the advantages of a high energy-conversion rate, environmental protection, and low noise and thus have attracted extensive research [1,2]. PEMFCs mainly comprises a proton exchange membrane, catalytic layer, bipolar plate (BPs), and gas diffusion layer (GDL). As one of the essential components of a proton-exchange-membrane fuel cell, the bipolar plate plays the role of hydrothermal management, conducting current between single cells, transporting gas, etc. Hence, it has an important impact on the performance of a PEMFC [3,4]. In addition, BPs can account for 30% of the cost and 80% of the mass of a fuel cell stack [5]. Due to the role of BPs in fuel cells, higher electrical conductivity and mechanical properties, lower hydrogen permeability, and interface contact resistance with GDL are necessary for BPs.

According to different materials, BPs can be divided into graphite BPs, metal BPs, and graphite/resin composite BPs. Graphite BPs are the most mature class of BPs at present, with the advantages of good electrical conductivity and low contact resistance. However, graphite BPs have high processing costs and poor mechanical properties [6]. Metal BPs have good electrical conductivity, mechanical properties, and easy processing [7], while the durability of metal BPs in the fuel-cell environment is not good, and the metal ions generated by corrosion will damage the proton exchange membrane and reduce the life of other components of the PEMFC. Therefore, additional costs are often required to prepare corrosion-resistant coatings to increase their durability [8,9,10,11]. The composite BPs are composed of graphite and resin, so the processing cost is lower, and their mechanical properties are also better than pure graphite BPs [12]. The fly in the ointment is that the electrical conductivity of the composite BPs is lower than that of graphite due to the presence of the non-conductive resin region, and it is difficult to achieve a balance between mechanical strength and electrical conductivity.

Many studies have been carried out to improve the performance of composite bipolar plates. Dong [13] used a modified epoxy resin to prepare a composite bipolar plate, which improved the interfacial compatibility of resin and graphite and improved the performance of the composite bipolar plate. Fengjing [14] introduced cactus-like carbon nanofibers into the composite bipolar plate by surface treatment, which improved the performance of the composite bipolar plate. Its electrical conductivity could reach 198.27 S/cm, and the contact resistance was reduced to 25.4 mΩ·cm^2^. R.B. Mathur et al. studied the effect of different conductive fillers on the performance of composite bipolar plates and found that carbon fibers, carbon nanotubes, and carbon black all play a positive role in the performance of composite bipolar plates [15]. BinHU et al. used graphite and NH4HCO3 to prepare a composite bipolar plate with a three-dimensional conductive network, which improved the electrical conductivity and the power density of proton-exchange-membrane fuel cells. The highest in-plane conductivity is 212.64 S/cm [16].

In fact, since the conductive paths inside the composite bipolar plate are often blocked due to the inhomogeneity of the material and the addition of resin, which will reduce the conductive performance of the composite bipolar plate, Therefore, a reasonable construction of conductive paths to ensure that the conductive paths inside the material are not blocked has also been proven to be an effective method to improve performance [17,18,19,20,21]. For example, Fan [22] uses the reasonable distribution of flake graphite (FG) and expanding graphite (EG) to construct a conductive path to improve the performance of the composite bipolar plate, and its conductivity can reach up to 332.64 S/cm. Additionally, the addition of conductive fillers can also effectively improve the electrical conductivity of composite bipolar plates [23,24,25,26]. At present, a variety of fillers have been proven to play a positive role in the performance of composite bipolar plates, such as carbon fibers (CF), multi-walled carbon nanotubes (MWCNTs), graphene, and conductive carbon black [27,28,29,30]. However, although nanoscale conductive fillers have good intrinsic conductivity, they tend to agglomerate in composites due to their excessively high surface energy, which will lead to reduced performance improvement and even the formation of regional agglomerated structures and will destroy the overall structure of composite BPs, resulting in performance degradation [31,32,33,34].

Chemical vapor deposition (CVD) is the mainstream process for preparing MWCNTs in large quantities, and the prepared MWCNTs have a high degree of graphitization. However, since MWCNTs are easy to agglomerate, adding the prepared MWCNTs to the graphite/resin mixed powder to prepare BPs can easily lead to the agglomeration of MWCNTs into particles. This will result in the inability of MWCNTs to play a role in improving performance [35,36]. However, by dispersing the catalyst particles on the CVD precursor by a suitable method, the uniform dispersion of the deposited product can be achieved, thereby avoiding agglomeration [37,38,39,40]. Therefore, the innovative in situ CVD method can avoid the agglomeration of nanofillers compared with the previous process using nanofillers to modify composite BPs. The MWCNTs without agglomeration can fully exploit their advantages in electrical and thermal conductivity, which is conducive to the further improvement of the performance of composite BPs.

This study successfully avoided this problem by adopting the in situ CVD method. The readily dispersible catalyst particles were uniformly dispersed on the surface of the graphite substrate in advance before deposition. Then CVD was performed to prepare MWCNTs directly on the surface of the graphite substrate. Since the catalyst particles have been dispersed in advance, the prepared MWCNTs also inherited this feature. In the composite BPs prepared by this method, the internal MWCNTs were dispersed together with the graphite particles, forming a uniform conductive path, which improved the conductivity of the composite BPs and reduced the contact resistance with GDL. At the same time, the in situ deposited MWCNTs also filled the structural defects of graphite particles and reduced the porosity and defects of composite BPs. Therefore, their mechanical properties were further improved, and can assist in expanding graphite and flake graphite together to build a conductive network. In this study, the process of modifying the properties of composite BPs was also compared with the current mainstream method of the direct addition of CNTs based on previous studies.

## 2. Experimental

### 2.1. Materials

The following raw materials were used in the preparation of in situ deposited carbon nanotubes: absolute ethanol (99.5%), cobalt nitrate hexahydrate (99%), high-purity nitrogen (99.9%), high-purity hydrogen (99.9%), and high-purity acetylene (99.5%).

The following raw materials were used in the preparation of composite BPs: PEEK resin (M_w_ = 200,000, Victrex Ltd, Shanghai, China.), flake graphite (quality purity 99.9%, mesh = 100, Shanghai Aladdin Co., Shanghai, China), expanded graphite (Purity = 99.9%, mesh = 800, Shanghai Aladdin Co., Shanghai, China ), and commercial multi-walled carbon nanotubes(Purity > 98%, length = 60–200 nm, average diameter = 12 nm, Sigma Aldrich (Shanghai) Trading Co., Shanghai, China) for comparison.

### 2.2. Preparation of MWCNTs by In Situ Chemical Vapor Deposition

In the pretreatment process of chemical deposition, a certain amount of expanded graphite powder was weighed, and cobalt nitrate hexahydrate equivalent to 5% mass fraction of the graphite mass was fully mixed with graphite under ultrasonic conditions heated to 70 ℃ to accelerate the volatilization of alcohol, and when the solution appeared as a paste, the solution was dried in an oven for 8 h to obtain the expanded graphite mixed with cobalt nitrate.

The treated expanded graphite powder loaded with cobalt nitrate was placed in a vapor deposition furnace. Heating in two steps—in situ reduction and in situ chemical vapor deposition. After heating to the reduction temperature at 400 °C, hydrogen was introduced for reduction for a period of time so that the 2-valent cobalt was reduced by hydrogen to catalytic monolithic cobalt particles. A vapor deposition furnace was then heated to the deposition temperature. In order to study the morphology of the in situ deposited MWCNTs at different temperatures, the in situ deposited MWCNTs prepared at X degrees Celsius were labeled as MWCNTs_x_. Based on our previous experiments. MWCNTs prepared at 780~900 degrees Celsius were mainly studied and labeled CNT_750_, CNT_780,_ CNT_810,_ CNT_840_, CNT_870_ and CNT_900_. The bipolar plate group with the addition of in situ deposited carbon nanotubes was named I-CBPs; the group with direct addition of commercial carbon nanotubes for comparison was named C-CBPs, and the blank control group without any nano-conducting agent was named BC-BPs.

### 2.3. Preparation of Composite Bipolar Plates

**Figure 1:**
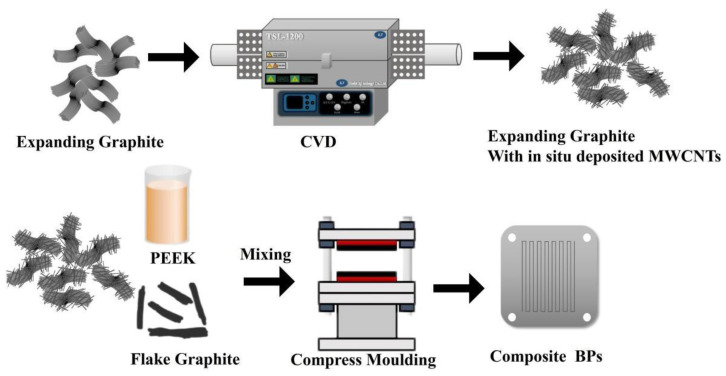
Schematic diagram of the preparation and structure of composite BPs.

The powder prepared in the previous step was mixed with resin and flake graphite. After the mixed powder was obtained, it was put into a mold for hot pressing. The mold used in this study was 40 mm × 40 mm in size, and the sample’s thickness depended on the powder’s amount. In order to meet the thickness requirements of the composite bipolar plate, its thickness was controlled at 0.4 mm, the molding temperature was controlled at 270 °C, and the molding pressure was 80 MPa. Each time it was molded, the pressurization–depressurization was carried out three times, which was used to ensure that the air inside the mold was utterly pressed out. The whole preparation process is shown in Figure 1.

### 2.4. Characterization of Deposition Products and Composite Bipolar Plates

Scanning electron microscopy (SEM, JSM−7600F, Japan Electronics Corporation, Tokyo, Japan) was used to study the morphologies of in situ deposited MWCNTs and the surface morphologies and cross-sectional morphologies of the prepared composite bipolar plates.

Transmission electron microscopy (TEM, FEI tecnai F20, FEI Corporation, Hillsboro, USA) was used to further study the microscopic morphology of the deposited MWCNTs, such as the number of wall layers and the diameter and to study the structure of the conductive network at the microscopic level.

Raman spectroscopy (Oxford MAX20, Hitachi, Ltd., Tokyo, Japan) were used to study the degree of graphitization of the in situ deposited MWCNTs and the number of graphite stacking layers.

A Rigaku X-ray Diffractometer Miniflex 600 (Japan Science Corporation, Tokyo, Japan) was used to test the X-ray diffraction patterns of MWCNTs, and the scanning range was 5~90°.

A Instron 3369 Universal Mechanical Tester (Instron Shanghai Ltd, Shanghai, China) was used to test the bending strength. The sample was shaped into a long strip with a size of 40 × 20 mm^2^ and measured by the three-point bending method, with a bending moment of 20 mm, and the bending strength was calculated from the bending stress. The bending strength was calculated using the following formula as Equation (1) shows:

(1) $$\varepsilon =6\frac{f\cdot h}{L^{2}}$$

(2) $$\sigma =\frac{3PL}{2bh}$$

where the displacement of the pressure head was taken as an approximation in this study; ***L*** was the span (mm); ***P*** was the bending loads (N), and ***b*** was the width (mm).

**Figure 2:**
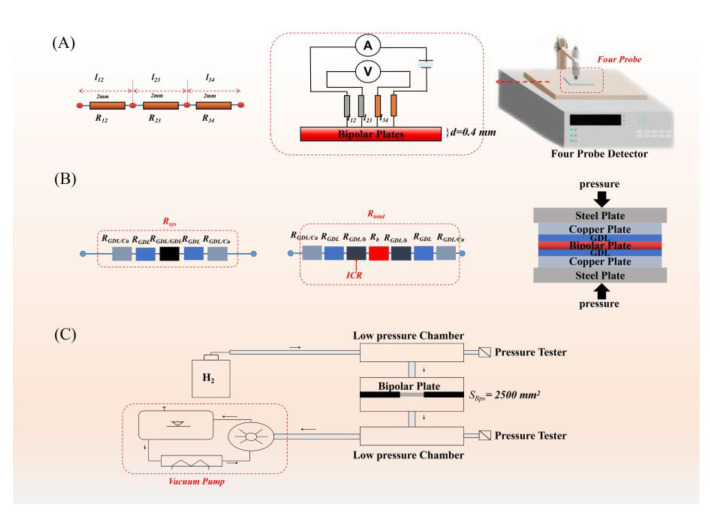
(**A**) Four-probe equipment and schematic diagram; (**B**) ICR testing equipment and schematic diagram; (**C**) hydrogen permeability testing equipment.

The conductivity of the composite bipolar plate was measured by the ST2258C four-probe method. The composite bipolar plate was prepared into a block sample of 40 mm × 40 mm. Its internal circuit and calculation formula were shown below. By dividing the measurement result by the thickness correction factor, the shape correction factor was obtained to calculate the conductivity of the composite bipolar plate. The conductivity test principle and equipment are shown in Figure 2A. The resistivity measured by the four-probe method can be calculated as shown in Equation (3) by the following equation.

(3) $$\sigma =\frac{I}{\pi dv}\ln(\frac{l_{13}l_{24}}{l_{12}l_{34}})$$

where ***I*** is the current, ***v*** is the voltage between point 2 and point 3, and d is the thickness of the BPs.

In order to obtain the hydrophobic performance of the composite bipolar plate, the JC2000A contact angle tester was used to test the contact angle of the composite bipolar plate, and the hydrophobic performance of the composite bipolar plate was obtained by calculating the angle between a single droplet and the surface of the composite bipolar plate.

The interface contact resistance (ICR) and area-specific resistance (ASR) measurement adopts the test standards in the DOE standard, and a FT361SJB ICR tester was used for testing ICR. The test principle and test circuit are shown in the Figure 2B, and the contact resistance between the composite bipolar plate and the GDL is calculated according to the following equation:

(4) $$R=\frac{V\cdot A_{s}}{I^{2}}$$

(5) $$R_{total}=2R_{GDL}+2R_{GDL}{}_{/Cu}+2R_{GDL/b}+R_{b}$$

(6) $$R_{\mathrm{sys}}=2R_{GDL}+2R_{GDL}{}_{/Cu}+2R_{GDL/\mathrm{GDL}}$$

(7) $$ICR{=R}_{\mathrm{GDL}/B}{=(R}_{\mathrm{total}}{-R}_{\mathrm{sys}}{-R}_{b})/2$$

(8) $$ASR=2ICR_{}+R_{b}$$

where ***R*** is the total contact resistance, ***V*** is the voltage drop through the setting, ***I*** is the applied current, and As represents the contact area of the sample. The test method is shown in Figure 2B, by placing two or three pieces of GDL into the test instrument, ***R_sys_***, ***R_GDL/GDL_*** can be measured, and and ICR can be obtained by Equations (4)–(8). ***R_total_*** is the tested resistance with the BPs, and Rsys is the tested resistance without BPs. ***R_GDL/Cu_*** is the interface resistance between copper plates and carbon papers, and ***R_GDL/b_*** is the ***ICR***. ***ASR*** can also be calculated.

The thermal conductivity of the samples was measured by hotdisk according to the international standard ISO 22007-2, using a Hot Disk TPS 2500S (Hot Disk Sweden, Stockholm, SWE) thermal conductivity measuring instrument

According to the DOE standard, a self-made device for testing the hydrogen permeability of the composite bipolar plate was made. A schematic diagram of the device is shown in the Figure 2C. By measuring the air pressure difference between the vacuum chamber and the gas chamber for a certain period of time, the hydrogen permeability rate was calculated by a formula.

In order to measure the electrochemical properties and corrosion resistance of composite bipolar plates, a Wavedriver100 Electrochemical Comprehensive Tester was used to test the corrosion resistance of composite bipolar plates. The corrosion resistance is characterized by measuring its tafel curve and calculating the corrosion current. According to the DOE standard, the specific parameters of the test are: conditions of 80 °C, 0.5 M H_2_SO_4_ and 2 × 10^−6^ M HF solution; the scanning range was from −0.5 V to 0.9 V, and the scan rate was 0.2 mV/s. The samples for the constant potential polarization method were tested at potentials of −0.1 V and 0.6 V, respectively, using the same solutions to simulate the positive and negative corrosion conditions of PEMFC. 

The Mike ASAP2460 Automatic Specific Surface and Porosity Analyzer BET (Micron Instruments, Inc. (Shanghai Branch), Shanghai, China) was used to analyze the pore size distribution, porosity, average pore size and other parameters of the composite bipolar plate. In order to reduce errors, the crushed small block samples were passed through multiple mesh screens. The error caused by the size is small. The test condition was 100 °C under a nitrogen atmosphere, and the degassing time was 8 h. In order to analyze the pore size distribution and the filling effect of nanofillers, the analysis was carried out by the DFT method.

## 3. Results and Discussion

### 3.1. Morphology of MWCNTs Prepared at Different Temperatures and Its Effect on the Properties of BPs

**Figure 3:**
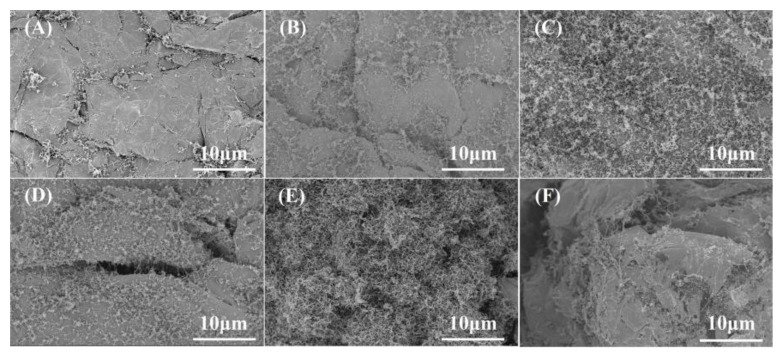
SEM images of MWCNTs prepared at different temperatures: (**A**) CNT_750_; (**B**) CNT_780_; (**C**) CNT_810_; (**D**) CNT_840_; (**E**) CNT_870_; (**F**) CNT_900_.

**Figure 4:**
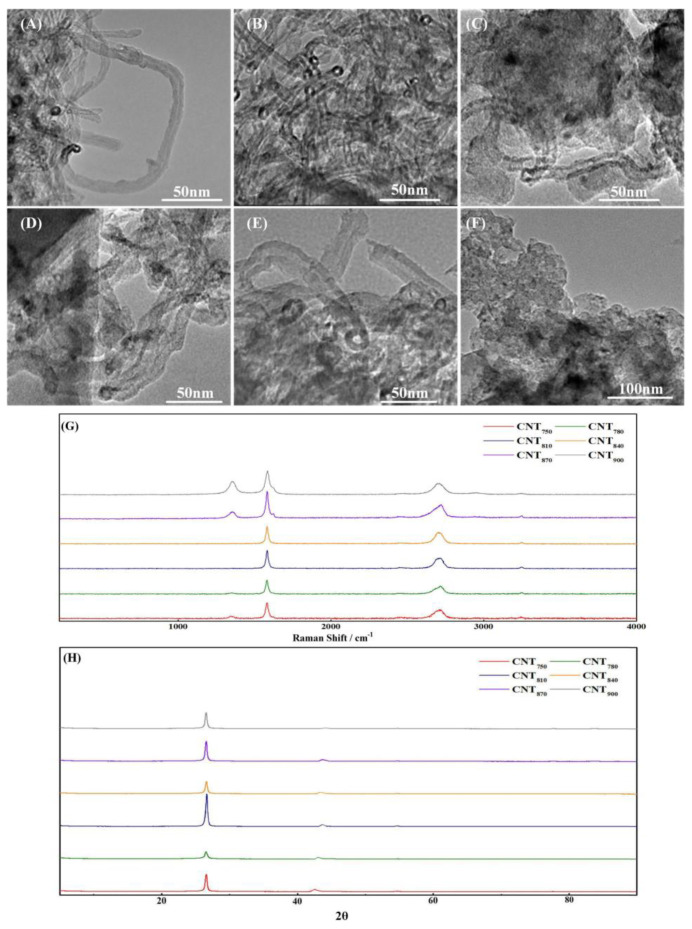
TEM images of MWCNTs prepared at different temperatures: (**A**) CNT750; (**B**) CNT780; (**C**) CNT810; (**D**) CNT840; (**E**) CNT870; (**F**) CNT900; (**G**) Raman spectra of MWCNTs prepared at different temperatures; (**H**) XRD of MWCNTs prepared at different temperatures.

To investigate the conditions under which the MWCNTs deposited in situ on EG can be uniformly dispersed on the EG surface to form a mesh-like conductive structure, the SEM images and TEM are shown in Figure 3 and Figure 4.

From SEM, the content of MWCNTs deposited on the EG surface was gradually increased with increasing deposition temperature, from sparse discrete MWCNTs at 750 °C and 780 °C, as Figure 3A,B shows; to a uniformly distributed network-like structure at 810 °C and 840 °C, as Figure 3C,D shows; and a sparsely encapsulated layer-like structure at higher temperatures, as Figure 3E,F shows. At lower temperatures, the dispersed metal particles did not agglomerate, but the reaction rate was also slower at this time, so the generated MWCNTs network was sparse. However, with further increase in temperature, the reaction rate was further accelerated, the mobility of catalyst particles was enhanced, and the diffusion rate of carbon atoms in the carbon source was accelerated, which led to the increase in the diameter of MWCNTs and the agglomeration on the EG surface and finally, the formation of loose carbon particles. 

After studying the macroscopic distribution of MWCNTs on EG, TEM was used to analyze the diameter, wall thickness and content of MWCNTs to further investigate the properties of in situ deposited MWCNTs. From the TEM results, the thickness of MWCNTs gradually became thicker with increasing temperature; the graphene layers on their tube walls gradually became indistinct, as Figure 4A–E shows, and finally formed carbon particles, as Figure 4F shows without lattice stripes.

According to the formation mechanism of MWCNTs, MWCNTs were formed through a dissolution–diffusion mechanism [41,42]. At low temperatures, the reaction location was limited by the diffusion rate of C elements, so the reaction can only occur on the side of the catalyst droplet, forming completely hollow MWCNTs. As the temperature increased, the diffusion rate of C atoms gradually increased and was able to react on all sides of the catalyst, which led to the gradual disappearance of the tube walls of MWCNTs with an oriented structure. The diameter also increases with the increase of the diffusion–precipitation region, finally leading to the transformation of MWCNTs into C particles.

Raman spectroscopy was used to investigate the graphitization of in situ deposited MWCNTs, and the results are shown in Figure 4G. The complete single-crystal graphite has only a sharp peak (g-peak) at 1580 cm^−1^, and the incomplete single-crystal graphite material has a peak (d-peak) at 1360 cm^−1^. The peak intensity indicates the number of non-graphitized boundaries in the material, that is, the turbostratic non-graphitized structure. Therefore, the ratio of peak heights can be used to characterize the degree of graphitization R (I_d_/I_g_) of carbon materials; the smaller R is, the higher the degree of graphitization is. The D-peaks of CNT_810_ and CNT_840_ were almost absent, which indicated that their graphitization was very high. 

However, it should be noted that the Raman spectrum is often used to determine the average value in a small range, and for the group with a small amount of deposition, the graphite of the substrate may have a certain impact on the results. The results are shown in Figure 4H. Except for the product at 900 degrees, the rest of the products had the characteristic peaks of MWCNTs (2θ ≈ 26°, 2θ ≈ 43°), which was also consistent with the TEM image results, because under TEM, the product at 900 °C was carbon particles, not typical carbon nanotubes.

**Figure 5:**
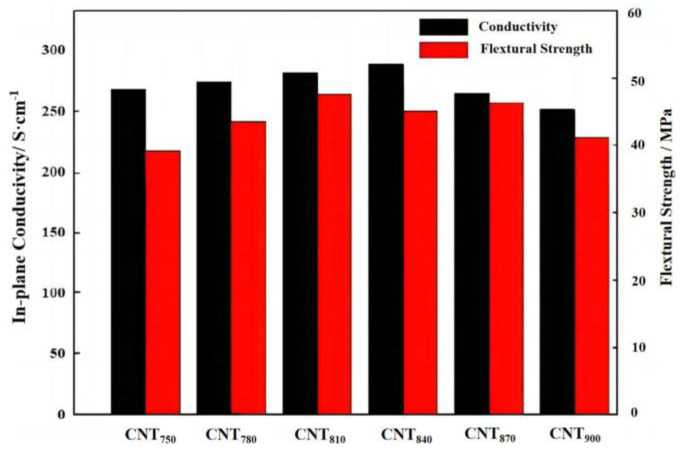
Electrical conductivity and flexural strength of composite BPs prepared from MWCNTs at different temperatures.

The 2% content of MWCNTs prepared at different temperatures was mixed with the 20% mass fraction of PEEK and the 80% of FG pressed into bipolar plates, and their conductivity and flexural strength were tested, as shown in Figure 5. It can be found that different MWCNTs have different effects on the performance of BPs under the condition of controlling a certain amount of MWCNTs addition. The MWCNTs produced at lower temperatures were only produced at specific parts of the EG, and no conductive network structure was produced, so the performance of BPs was poorly improved. As the temperature increased, the conductive network was gradually improved, and conductivity was gradually constructed. It can be found from the SEM image that the BPs filled the defects of graphite, such as the seams and holes of EG, so the conductivity of BPs was improved. The bending strength also has a certain improvement effect. When the temperature rose to a very high level, not only the graphitization decreased but also the formation of loose carbon particles did not improve the performance of BPs, so the bending strength and conductivity were lower. From the results of the experiments, the CNT_810_ were the most useful for the improvement of the properties of BPs, so the CNT_810_ were used for the subsequent experiments.

### 3.2. Effect of the Ratio of FG and EG on Structure and Properties of BPs

**Figure 6:**
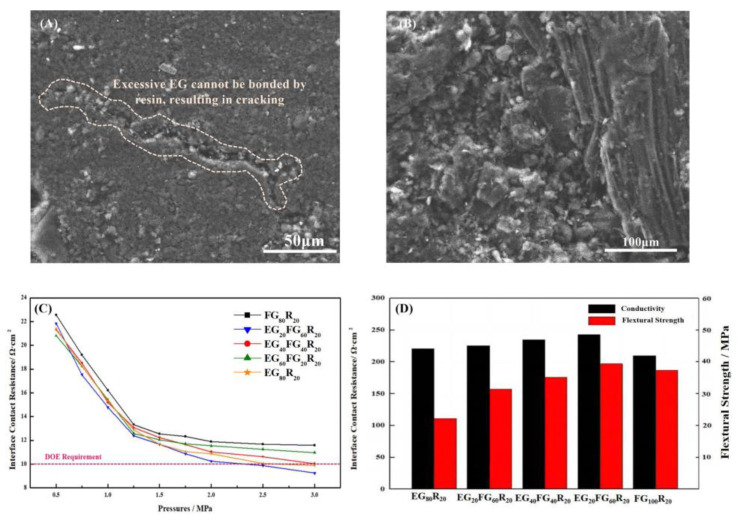
(**A**,**B**) SEM images of the composite BPs prepared by EG and FG; (**C**) effect of different ratios of EG and FG on ICR; (**D**) effect of different ratios of EG and FG on the performance of BPs.

Different ratios of EG and FG can construct different structures of conductive pathways. In this subsection, the effects of different ratios of EG and FG on the structure and properties of BPs were investigated. Different proportions of EG and FG powders were mixed with resin and pressed into BPs, and the samples were named as shown in Table 1. The planar conductivity, flexural strength and contact resistance of the pressed BPs were tested, and the results are shown in Figure 6.

EG and FG have different morphologies and therefore have different effects on the structure of BPs: EG is an excellent thermal and electrical conductivity material, but because of its high porosity and low strength [43,44,45], too much EG in the composite BPs was conducive to the rise in electrical conductivity but led to a decline in flexural strength. At the same time, the small mesh size of EG was often difficult to form into a dispersion of the conductive channel because of agglomeration, and this led to cracks, as shown in Figure 6A. It is necessary to add a large mesh size with a large area ratio of laminated graphite to improve the bending strength and to improve the construction of the conductive channel to form the structure shown in Figure 6B. The conductivity of the bipolar plate can reach 289.37 S/cm, which is 7.6% higher than the 268.74 S/cm of the pure EG bipolar plate. The ICR with GDL was also reduced, as shown in Figure 6C,D, thanks to the graphite forming a complete conductive pathway. The increase in flexural strength was even greater, from 22.23 MPa to 39.37 MPa compared to pure EG. Compared to pure EG bipolar plate, there was a synergistic effect between FG and EG, which can form a more dispersed conductive pathway and therefore has a greater effect on the conductivity enhancement of BPs. Therefore, in the subsequent study, a resin ratio of FG:EG = 3:1 was used for further experiments. 

### 3.3. Effect of In Situ Deposited MWCNTs on the Structure and Properties of BPs

**Figure 7:**
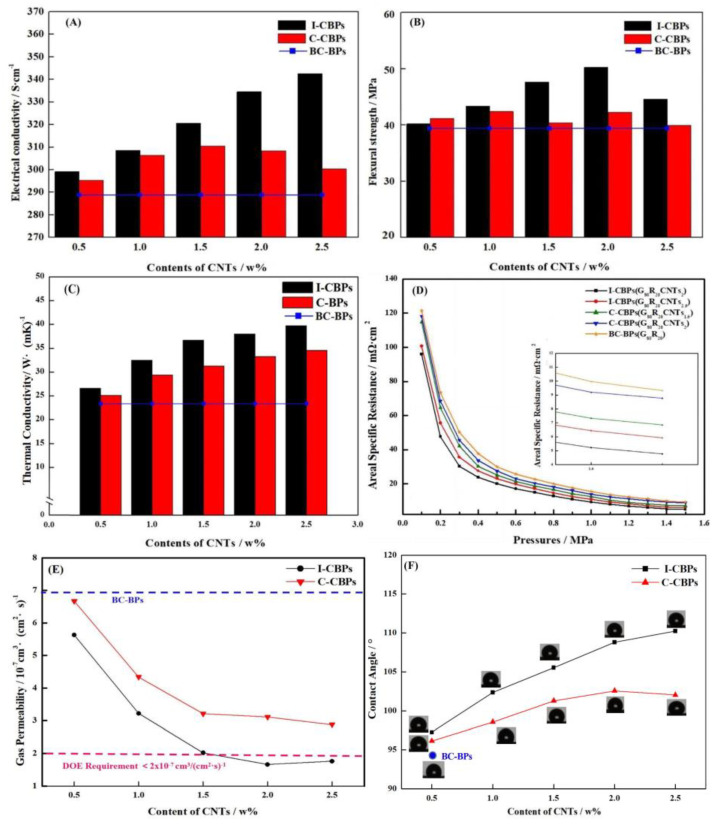
Effect of MWCNTs content and addition method on the performance of BPs: (**A**) in-plane conductivity; (**B**) flexural strength; (**C**) thermal conductivity; (**D**) area-specific resistance; (**E**) gas permeability; (**F**) contact angle.

**Figure 8:**
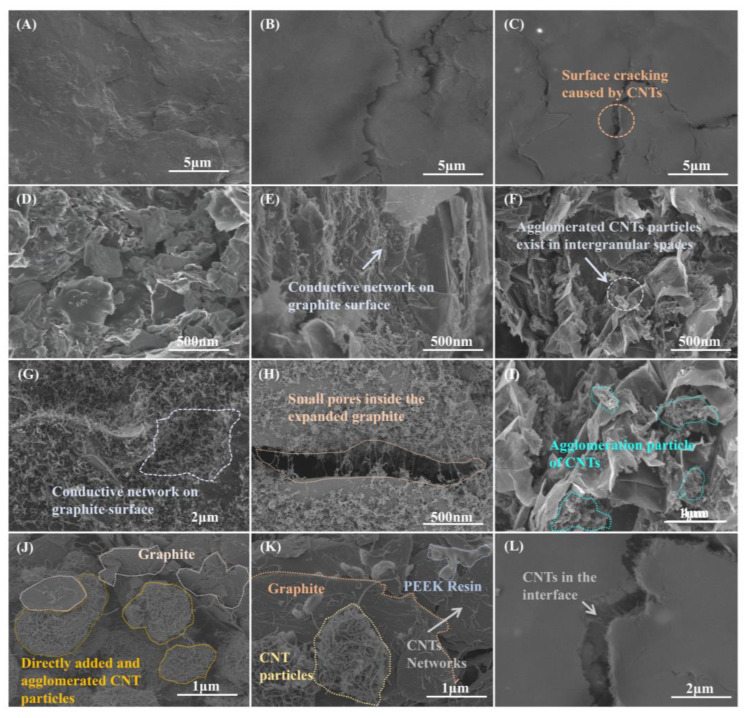
Surface morphology of composite bipolar plate: (**A**) BC-BPs; (**B**) I-CBPs; (**C**) C-CBPs; FESEM diagram of composite bipolar plate cross-section: (**D**) BC-BPs; (**E**) I-CBPs; (**F**) C-CBPs; (**G**) In situ deposited MWCNTs formed a network-like structure; (**H**) in situ deposited MWCNTs filled the pores of EG; (**I**) excess in situ deposited MWCNTs agglomerated inside BPs; (**J**,**K**) agglomerated MWCNTs particles inside C-CBPs; (**L**) surface morphology of BPs with crack.

Although the reasonably proportioned EG/FG has much-improved performance compared to the pure EG plate, there are still many defects and porosity and shortcomings in performance, as shown in Figure 6C. The ICR of any group of samples had difficulty in meeting the DOE requirement of 2025 (pressure = 1.5 MPa). MWCNTs, as nanomaterials, have high surface energy and are prone to agglomeration, while the in situ deposition of MWCNTs using catalyst particles already dispersed on the surface of graphite particles can effectively improve the agglomeration phenomenon as described in the previous section. Different ratios of in situ deposited MWCNTs and commercial MWCNTs added directly as a comparison were used to prepare BPs, and the samples are shown in Table 2. The control samples with the direct addition of commercial MWCNTs were named D-CBPs and with in situ deposited MWCNTs were named I-CBPs, and the BPs without any conductive filler added to the blank control group were named BC-BPs (Sample EG_20_FG_60_R_20_). The specific groups are shown in Table 2 below. The performance test of composite BPs is shown in Figure 7 and the FESEM images of the surfaces and sections of the composite BPs of different groups are shown in Figure 8. The specific performance values of all composite BPs are shown in Table 3.

The results of conductivity tests in Figure 7A show that the conductivity of I-CBPs increased with the addition of MWCNTs. This was due to the high intrinsic conductivity of MWCNTs and their ability to form conductive pathways inside the BPs. As shown in Figure 8E–G, MWCNTs covered the graphite surface and completed the conductive network. MWCNTs formed a network structure in the surface layer of EGs, which expanded the contact surface between conducting particles in BPs and filled the gaps between particles inside BPs as Figure 8H shows, thus contributing to the formation of a more complete and integral conducting network of FG/EGs, which effectively improved the electrical conductivity. Compared with the BC-BPs without any MWCNTs added as a conductive filler (289.37 S/cm), the conductivity was 334.57 S/cm at 2% addition and 342.49 S/cm at 2.5% addition for I-CBPs. The conductivity of C-CBPs with the direct addition of MWCNTs showed an increasing trend followed by a decreasing trend. This was because, although the direct addition of MWCNTs, followed by mechanical dispersion, can form a conductive pathway inside the BPs, this dispersion effect was not sufficient to completely disperse the MWCNTs into a homogeneous conductive network and can not be totally wetted, as Figure 8F shows. When too many MWCNTs were added, agglomerated micron-sized particles were formed, as shown in Figure 8J,K, which were less compatible with the resin and had a looser internal structure; instead, it left a lot of pores and gaps, thus having a negative effect on conductivity enhancement [46,47].

The results of the flexural strength tests of BPs are shown in Figure 7B. For I-CBPs, the flexural strength rose with low CNT content and decreased with high CNT content, and the best flexural strength was achieved under the condition that the mass fraction of MWCNTs was 2%, and its flexural strength could reach 50.24 MPa. MWCNTs as nano-fillers have a good strengthening effect on EG, and the surface-grown MWCNTs can not only make the contact points between graphite particles increase and make the graphite surface rougher [48] but also fills the pores of the matrix [49]. CNTs with the right content have a good strengthening effect on EG. However, this improvement effect is not unlimited; for I-CBPs, the excessive addition of in situ deposited MWCNTs will still form agglomeration, although the tendency is lower compared to C-CBPs, and the excessive CNTs were difficult to be wetted, thus generating a large number of voids and air residue, which was unfavorable to the improvement of the mechanical properties of composite BPs [50,51]. As shown in Figure 8F,I, the cross-sectional SEM image of BPs shows the particles formed by the agglomeration of MWCNTs; this agglomeration of brittle large particles for the stress-bearing effect is completely inferior to the uniformly dispersed network of MWCNTs. For C-CBPs, with low MWCNT content, MWCNTs could be dispersed inside the material, so the mechanical properties increased, but as the MWCNT content increased, MWCNTs agglomerate into particles. As the CNT content rose to a threshold value, the mechanical properties of the material decreased, which was consistent with previous reports in the literature [52]. CNTs that underwent agglomeration not only had poor mechanical properties but also poor compatibility with the resin phase, which led to an increase in defects and a decrease in flexural strength.

The results of the thermal conductivity measurement by hotdisk are shown in Figure 7C. The results are similar to those of the conductivity test, but the difference is that the intrinsic thermal conductivity was high for both the deposited CNTs and the directly added CNTs, so the improvement effectiveness for the thermal conductivity of the composite BPs was huge. Compared with BC-BPs, the introduction of small amounts of CNTs as secondary fillers can improve the electrical conductivity of carbon fiber or graphite-filled polymers. This was due to the small size, high aspect ratio and low permeation threshold of carbon nanotubes. CNTs can form secondary permeation networks and also act as bridges and fill small gaps between larger primary fillers, enhancing the thermal conductivity of composite BPs [53,54,55].

In order to investigate the effect of in situ deposited MWCNTs on the surface morphology and contact resistance of BPs in more depth, several groups of BPs with good overall performance were selected for ASR tests with different pressures, as Figure 7D shows. The ASR mainly includes the conductivity of the composite BPs themselves and the ICR with GDL. Previous studies have shown that MWCNTs had a positive effect on the conductivity of composite BPs, and, although the addition of MWCNTs caused the graphite part of the surface of the composite BPs to become rough, the ASR was tested under pressurized conditions, when the surface-dispersed reticular high-conductivity MWCNTs will fully play a role in reducing the ICR with GDL. For C-CBPs, the conductive effect of agglomerated CNTs was weakened, so the improvement was not as good as that of I-CBPs. As the content of CNTs increased, more and more CNTs were difficult to be wetted, which also had a negative effect on ASR [56]. On the other hand, molded composites often have an enriched layer of resin on the surface due to extrusion [57], which is extremely detrimental to ASR. In the composite BPs with CNTs added, the CNTs moved to the surface of the composite BPs with the resin, which also led to the decrease in the ASR of the composite BPs, as shown in Figure 8C,L.

**Figure 9:**
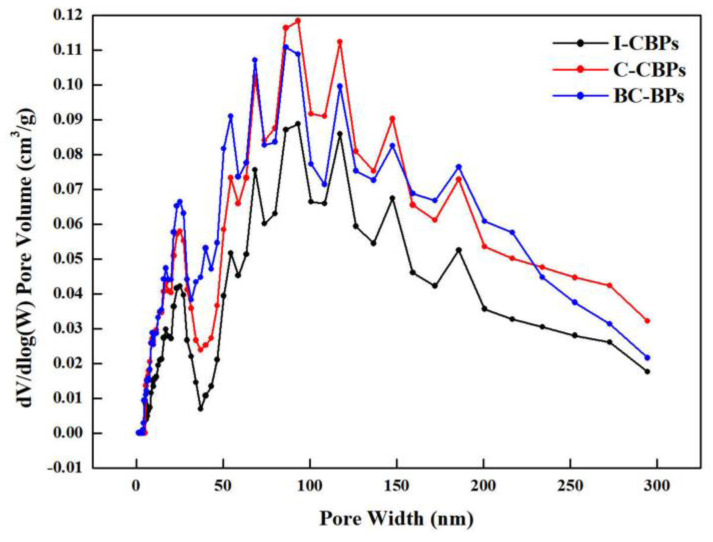
Results of BET DFT and the filling effect of different morphologies of MWCNTs on the pore space.

The hydrogen permeability of the composite BPs is shown in Figure 7E. In order to investigate more deeply the filling effect of different forms of MWCNTs on the pores of the composite BPs and the mechanism of improving the gas tightness, I-CBPs-G_80_R_20_CNT_2_ and C-CBPs-G_80_R_20_CNT_2_, BC-BPs were selected for nitrogen adsorption–desorption tests, and the porosity was calculated according to the DFT method using the adsorption–desorption curve. The results are shown in Figure 9. The results show that the gas permeability of I-CBPs was lower and continues to decrease with increasing CNT content and was lower compared to C-CBPs, and the hydrogen permeability can meet the DOE requirements when the filling amount is greater than or equal to 2%. Combined with Figure 9, it can be found that the in situ deposited CNTs filled the pores well at all porosities, while C-CBPs had a better filling effect for large pores above 150 nm but almost no filling effect at all for pores between 50 and 100 nm. This may be because, compared with I-CBPs, the CNTs inside C-CBPs may be due to agglomeration, which makes it difficult to fill the surface of EG particles and the tiny pores between EG, FG and resin, but a small number of MWCNTs were still dispersed during the preparation process, so they can play a similar filling role as I-CBPs for the pores between 0~50 nm [58,59,60]. Therefore, the pores between 0~50 nm can be filled similarly to I-CBPs, although the effect is not as good as that of I-CBPs.

Hydrophobicity has a significant impact on the role of BPs in playing a key role in hydrothermal management. As mentioned earlier, for I-CBPs, the MWCNTs deposited in situ filled the pores of EGs well, thus making the surface more structurally complete with fewer defects. This led to the improvement of the hydrophobicity of BPs, but the opposite effect was observed for agglomerated MWCNTs. As the content of MWCNTs gradually became higher, it can reach 110.24°, as Figure 7F shows. As discussed in the ASR results, MWCNTs were able to be on the surface of composite BPs, but, due to compatibility and inability to be wetted, it reduced ASR and increased the conductive pathway on the surface; on the other hand, excessive MWCNTs led to cracks and defects on the surface. This was illustrated by the presence of a large number of MWCNTs in the cracks, as Figure 8C,L shows. For C-CBPs, the agglomeration is stronger and the dispersion was lower, so the improvement of the hydrophobicity of BPs was not as good as that of I-CBPs. In addition, it was shown that the increase in hydrophobicity was also related to the increase in the surface roughness of composite BPs and the increase in the exposure rate of graphite [60], while the SEM images of composite BPs showed that the surface of graphite deposited with CNTs had a fluff-like structure, which could improve the surface roughness of graphite.

**Figure 10:**
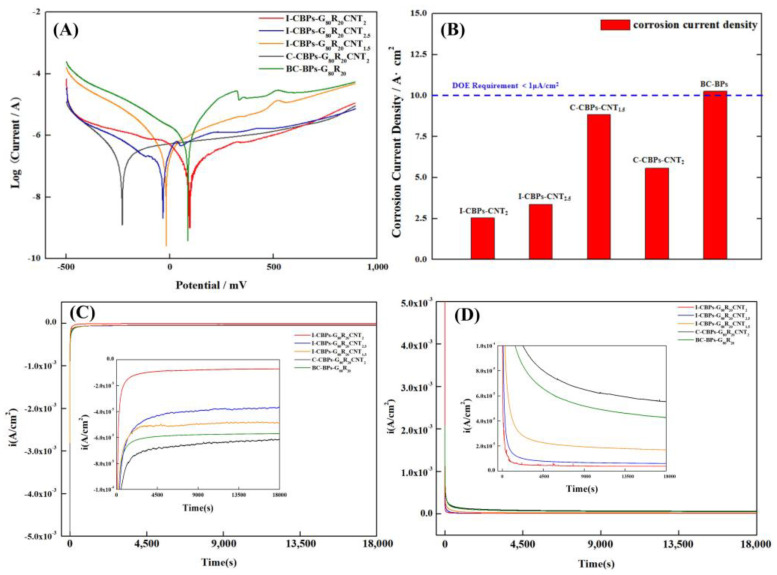
(**A**) Tafel curves of composite BPs; (**B**) corrosion current density of composite BPs; (**C**) constant potential polarization method to simulate PEMFC cathodic environment; (**D**) constant potential polarization method to simulate PEMFC anodic environment.

Based on the previous ASR tests, the samples were tested for electrochemical corrosion, and the corrosion currents of the different samples were derived from Tafel curves, as Figure 10A,B shows. Due to the high corrosion resistance of the selected PEEK resin, all parts had good corrosion resistance. From the results of the corrosion resistance tests, I-CBP and C-CBP were found to meet DOE requirements. Since the corrosion current density was calculated using the surface area of the sample as a constant, when there are many defects and cracks on the surface of the sample, the actual contact area between the sample and the corrosion solution increases, resulting in an increase in the corrosion current [61,62]. The in situ deposited MWCNTs filled the defects and thus reduced the corrosion current density, while the agglomerated particles of C-CBPs are inherently porous and sparse in structure, which also led to an increase in the porosity of BPs and thus had less effect on reducing the corrosion current density than I-CBPs. As discussed in the chapter on hydrophobicity, MWCNTs have both a filling effect on the surface of the composite BPs and may increase their surface defects, the actual effect depending on which one prevails. So the trend of their corrosion current also decreased first and then increased with the increase in MWCNT content. The results of the constant potential polarization tests, as Figure 10C,D shows, also shows that the in situ deposited CNTs favored the reduction of corrosion currents in both cathodic and anodic simulated environments due to the dominant filling effect, while the direct addition of commercial CNTs accelerated corrosion, which is also consistent with previous reports in the literature. The test results in the constant current method show that the effect of increasing corrosion current dominated when the content of CNTs exceeded 2% in the C-CBPs group, so the corrosion resistance decreases continuously, as the content of CNTs continued to rise, and this trend of decreasing corrosion current first as the filler increases was also consistent with the previous literature studying the corrosion effect of bipolar plates [61,62,63]. In summary, thanks to the chemical inertness of PEEK resin and carbon materials, all samples showed good stability in the PEMFC simulation environment with pH = 1, and their lower corrosion current densities indicated that the materials were in the operating environment of PEMFC. It has good chemical stability and will not corrode because the pH value is too low.

## 4. Conclusions

The purpose of this study is to construct composite BPs with uniform conductive networks using in situ deposited MWCNTs assisted by FG and EG. The conductive pathways were constructed by reasonably controlling the ratio of EG and FG, and the BPs were enhanced by in situ deposited MWCNTs. Due to the advance dispersion of catalyst particles for deposition and controlled reaction temperature, uniformly dispersed network-like MWCNTs were obtained, which could play the role of filling pores and reducing micropores in the composite BPs, making the conductive network structure more perfect, so that highly conductive, high-strength and corrosion-resistant BPs can be obtained. Thanks to the characteristics of the CVD method, the deposited MWCNTs exhibited a uniform network-like structure and were dispersed on the surface of the graphite particles and within the defects. For the best I-CBPs-G_80_R_20_CNT_2_, the conductivity can reach 334.57 S/cm and the flexural strength 50.24 MPa. The corrosion resistance, hydrogen transmission rate, contact resistance and corrosion current density can meet the DOE standard by 2025. In addition, since in situ deposited MWCNTs are uniformly dispersed, only a small amount of addition is required to meet the demand, and the CVD method is one of the mainstream methods for preparing MWCNTs, so it is one of the candidates for composite BPs that can be produced in batches. Compared with the previously used method of the direct addition of CNTs, the in situ deposition of CNTs by CVD is not only beneficial to the performance of composite BPs and avoids the agglomeration problem often faced by nanofillers but also can effectively reduce the cost of composite BPs.

## Figures and Tables

**Table 1:** Ratio(wt%) of EG, FG and resin for different samples.

Sample	Expanded Graphite wt%	Flake Graphite wt%	Resin wt%
FG_80_R_20_	0%	80%	20%
EG_20_FG_60_R_20_	20%	60%	20%
EG_40_FG_40_R_20_	40%	40%	20%
EG_60_FG_20_R_20_	60%	20%	20%
EG_80_R_20_	80%	0%	20%

**Table 2:** Ratio(wt%) of graphite and MWCNTs for different samples.

Groups	Sample	Graphite wt%	Resin wt%	In Situ Deposited MWCNTs wt%	Commercial MWCNTs wt%
I-CBPs	G_80_R_20_CNT_0.5_	80%	20%	0.5%	0
	G_80_R_20_CNT_1_	80%	20%	1%	0
	G_80_R_20_CNT_1.5_	80%	20%	1.5%	0
	G_80_R_20_CNT_2_	80%	20%	2%	0
	G_80_R_20_CNT_2.5_	80%	20%	2.5%	0
C-CBPs	G_80_R_20_CNT_0.5_	80%	20%	0	0.5%
	G_80_R_20_CNT_1_	80%	20%	0	1%
	G_80_R_20_CNT_1.5_	80%	20%	0	1.5%
	G_80_R_20_CNT_2_	80%	20%	0	2%
	G_80_R_20_CNT_2.5_	80%	20%	0	2.5%
BC-BPs	G_80_R_20_	80%	20%	0	0

**Table 3:** Performance of composite BPs.

Groups	Sample	Electrical Conductivity /S·cm^−1^	Flexural Strength /MPa	Thermal Conductivity /W·(mK)^−1^	Hydrogen Permeability /10^−7^cm^3^·(cm^2^·s)^−1^	Contact Angle
I-CBPs	G_80_R_20_CNT_0.5_	298.88	40.26	26.62	5.63	97.25
	G_80_R_20_CNT_1_	309.57	43.37	32.48	3.22	102.37
	G_80_R_20_CNT_1.5_	322.16	47.25	36.65	2.02	105.56
	G_80_R_20_CNT_2_	334.57	50.24	38.06	1.66	108.79
	G_80_R_20_CNT_2.5_	342.49	44.53	39.72	1.76	110.24
C-CBPs	G_80_R_20_CNT_0.5_	296.53	41.03	25.1	6.67	96.14
	G_80_R_20_CNT_1_	306.62	42.36	29.36	4.34	98.56
	G_80_R_20_CNT_1.5_	310.27	40.56	31.25	3.21	101.27
	G_80_R_20_CNT_2_	308.54	42.28	33.26	3.11	102.56
	G_80_R_20_CNT_2.5_	300.10	39.86	34.57	2.88	102.03
BC-BPs	G_80_R_20_	289.37	39.37	24.25	6.93	94.47

## Data Availability

Data available on request due to restrictions, e.g., privacy or ethical. The data presented in this study are available on request from the corresponding author.
